# Recruitment of the motor system during music listening: An ALE meta-analysis of fMRI data

**DOI:** 10.1371/journal.pone.0207213

**Published:** 2018-11-19

**Authors:** Chelsea L. Gordon, Patrice R. Cobb, Ramesh Balasubramaniam

**Affiliations:** 1 Cognitive & Information Sciences, University of California, Merced, California, United States of America; 2 Psychological Sciences, University of California, Merced, California, United States of America; University of Western Ontario, CANADA

## Abstract

Several neuroimaging studies have shown that listening to music activates brain regions that reside in the motor system, even when there is no overt movement. However, many of these studies report the activation of varying motor system areas that include the primary motor cortex, supplementary motor area, dorsal and ventral pre-motor areas and parietal regions. In order to examine what specific roles are played by various motor regions during music perception, we used activation likelihood estimation (ALE) to conduct a meta-analysis of neuroimaging literature on passive music listening. After extensive search of the literature, 42 studies were analyzed resulting in a total of 386 unique subjects contributing 694 activation foci in total. As suspected, auditory activations were found in the bilateral superior temporal gyrus, transverse temporal gyrus, insula, pyramis, bilateral precentral gyrus, and bilateral medial frontal gyrus. We also saw the widespread activation of motor networks including left and right lateral premotor cortex, right primary motor cortex, and the left cerebellum. These results suggest a central role of the motor system in music and rhythm perception. We discuss these findings in the context of the Action Simulation for Auditory Prediction (ASAP) model and other predictive coding accounts of brain function.

## Introduction

In the case of (most) music, we do not merely passively receive temporal patterns, but actively engage with the sound stream by discerning an underlying periodicity. This profound shaping of temporal perception is central to understanding and participation in music, dance and even speech/conversation. In recent years, neuroimaging studies have shown that passively listening to music activates brain regions that reside in the motor system proper. The same neural correlates underlying the creation of music and moving to music appear to be involved even when one is only listening to a musical piece [1–7].

The motor system has received increasing attention in non-purely-motor domains [8–12]. Activity in motor regions during perception of human actions and language is ubiquitous. In early theories of cognitive processing, motor processes and perceptual processes were understood as entirely separate and encapsulated mechanisms [13]. As evidence accumulates that shows this is not the case and there is substantial overlap among the domains, theories of action production and action perception must be informed accordingly. More recent proposals argue for common coding of perceptual and motor information [14, 15] that arises primarily due to the co-activation of perceptual and motor components of a given action. Anderson’s [16] theory of neural reuse additionally suggests that we should expect newly evolved functions such as language to make use of previously instantiated neural mechanisms whose computational functionality can be co-adapted for new purposes. As such, it is likely that motor activation observed during speech perception, for instance, corresponds to a sharing of computational or functional resources for perception and production of a given speech sound. We can expect a similar sharing of resources for music production and music perception as well.

Patel and Iversen [17] advanced a theory of motor activation during music perception called the ASAP (Action Simulation for Auditory Prediction) hypothesis. The theory suggests that the same neural underpinnings involved in the simulation of body movements are utilized by the motor planning system to entrain neural activation with musical beat. This entrainment allows simulations to be used online during music listening as a predictive sensory signal for the upcoming music beat. The simulation is not tied to a particular effector-based movement, but a simulation of a timed, rhythmic motion. Patel and Iversen suggest the dorsal auditory stream as a potential underlying neural pathway for this process [18].

Rauschecker [19] has also proposed that a unified function of the dorsal stream may be anticipatory control of sensorimotor events. In particular, he suggests the posterior superior temporal (ST) regions, along with the inferior parietal lobe (IPL), interface extensively with PMC, linking the motor and auditory regions to established sensorimotor networks for audiomotor processes, such as speech and music. This network is established through similar mechanisms to those used in motor control theories [20, 21], where a feed-forward projection carrying an efference copy of a motor command is used as a prediction of the upcoming sensory consequences, which can then be compared with the actual sensory outcome of the motor act. Rauschecker proposes that the projection from inferior frontal gyrus (IFG) to vPMC is a likely candidate for carrying an efference copy, while IPL to posterior ST would carry an “afference” copy of the predicted motor signal, thus allowing a continuous audiomotor predictive loop underlying smooth perceptuomotor abilities.

Another candidate theory, suggested by Schubotz [11], is of active sensory prediction of events using the motor system. Schubotz extends the idea of emulators from motor control theory to encompass inanimate event perception in addition to human action prediction. Schubotz suggests the following: when we repeatedly hear a melody, the lateral PMC builds up sensorimotor representations using input from association areas of the cortex. These sensorimotor representations are only audiomotor, lacking the proprioceptive-motor representations that are involved in an actual performed movement. The lateral PMC eventually establishes an internal model of this melody which can be used for perceptual prediction. This internal model is similar to that involved in motor control, but with the components for movement and sensory feedback removed. Schubotz proposes what she calls the HAPEM (Habitual Pragmatic Event Map) framework, which states that “the prediction of an event that is structured with regard to a property P engages the area of the lateral premotor cortex that is best adapted to specify its motor output in terms of property P”. What this means is that the perception of events with different properties recruits particular somatotopic regions of vPMC, selected based on similarity to the underlying properties of that area of vPMC. For instance, the regions of vPMC that correspond to executing and observing mouth movements are recruited for the perception of rhythmic events, due to the underlying rhythmic nature of the vocal system.

The above theories all posit that cortical motor areas play a role in music listening. Another emerging theme is that of the motor system having a predictive role in perceptual processes. These accounts are primarily in agreement in terms of which sub-areas in the motor system are involved. Schubotz’s framework directly proposes involvement of both lateral PMC and pre-SMA/SMA, while ASAP and Rauschecker’s theory both set the dorsal auditory stream (which includes dPMC) as the primary substrate. However, activated regions within the motor system measured by neuroimaging methods tend to vary between research studies. For instance, numerous music listening experiments report motor activity in both supplemental motor area (SMA) and dorsal premotor cortex (dPMC) [1– 6]. Among these studies, a few show neural activations in cerebellum [2, 4, 5] or primary motor cortex (M1) [7] during a music listening task. Said differently, most music-listening studies do not show activation in every region of the motor system, nor do they show uniform activation in any one part of the motor system. In order to gain insight into the functional contribution of the motor system to passive music perception, one necessary step is to determine which motor regions are consistently contributing across music listening instances.

There are many factors likely to contribute to differences across studies, as each individual experiment has its own musical stimuli that vary in terms of particular characteristics, such as rhythmicity, familiarity, and valence of the music, for instance. Stimuli consisting of highly regular rhythmic structure might engage brain regions important for timing and sequential structure (i.e., supplementary and pre-supplementary motor areas and the cerebellum), while others might not. Experiments also vary in terms of what a participant is directed to focus on in these paradigms, ranging from complete passive listening (not attending) to judging beat or other characteristics of the stimuli. Such task demands are also likely to influence which regions are active, as directing attention to a stimulus may encourage focusing on particular aspects of the music, such as its beat or rhythmicity. In the present study, we are interested in discovering what motor regions are engaged during all music perception—those activated during passive listening. We define passive listening as attentive listening while remaining still (i.e., not tapping along to the music).

Identifying which regions are active consistently across all music listening tasks would help gain insight into the underlying processes and hone existing theories. Many theories outlining the functional contribution of individual motor areas exist, which can be used to determine what particular function is being carried out in a task utilizing that motor region. If one critical component is the dorsal auditory stream, which has a proposed role in motor planning and mapping auditory information onto potential motor acts, we should observe observation in dPMC [22, 23]. If activation is found in vPMC, the underlying mechanism might be similar to that proposed in the action observation network, which is responsible for mirror system activity for observed and produced actions [24, 25, 26]. Many studies that involve music with beat manipulation report activity in SMA and pre-SMA regions, which are presumed to be important for sequential processing of action-related stimuli and for inhibition of movements, respectively [27, 28]. Thus, SMA activity might indicate processing of sequential aspects of the music, and pre-SMA the inhibition of the natural tendency to move or sway to the music. We also might observe activation of structures in the basal ganglia, which appear to be involved in beat perception [29]. The basal ganglia are important for movement timing and sequential movement execution [30, 31]. M1 activation corresponds to particular motor commands that are carried out by specific muscle groups [32] and has also been found active during observation of actions [33, 34, 35]. Finally, the cerebellum is known for its crucial role in motor timing and coordination. Research on sensorimotor adaptation has long focused on the role of the cerebellum in predicting sensory consequences of movement and adapting to errors in these predictions (for a more interactive view see [36, 37, 38, 39]). Furthermore, cerebellar activation in conjunction with hippocampal activity is thought to underlie spatiotemporal prediction of movements [40]. This implication in predictive processes of motor control might extend to imagined and simulated motor computations, e.g. the cerebellum might be active in musical prediction even when no direct motor control is required.

In order to determine which of these regions show reliable and consistent activation during music perception, we employed a meta-analysis of all neuroimaging experiments consisting of music listening using an activation likelihood estimation (ALE) [41]. We predict that from this meta-analysis will emerge a pattern of activation that will enlighten and instruct future theories aiming to explain the motor-specific contributions to passive music perception. Activation of any of the motor regions will provide conclusive evidence for the involvement of the regions of the brain typically considered “action areas”, in the perceptual domain of passive music listening. This will inform theories about what roles are played by the traditional motor system.

## Methods

Meta-analyses provide a formal, statistical integration to combine the results of several studies that address a set of related research questions. There are several methods available for the meta-analysis of neuroimaging data and careful consideration was given as to which was most appropriate for this study. First, our study aims were to synthesize neuroimaging data of studies comparing rest and passive listening. More specifically, we wanted to identify regions of consistent activation across studies. Activation likelihood estimation (ALE) meta-analysis [41] addresses this by treating the spatial relationship between within study foci as fixed effects and between study relationships as random effects. Secondly, we considered the characteristics of our dataset. Unlike some other methods (e.g., KDA and MKDA), ALE uses a Gaussian kernel. When several distinct foci are located within the same general area, the Gaussian kernel is most likely to recover the separate foci. And, in general, if the spatial error on peak locations is approximately Gaussian (a reasonable assumption), then the Gaussian kernel will likely yield the most sensitive results. To investigate our research questions, we conducted ALE meta-analysis. Imaging studies commonly report brain locations of task-induced activations as coordinates in 3D space (x,y, and z). ALE meta-analysis techniques can be used to identify reliable activation patterns in 3D space across studies. ALE is a coordinate-based approach to a meta-analysis, allowing researchers to integrate imaging data. Studies are collected, coded and interpreted using analytical methods to assess likelihood of activation through agreement or overlap in activation patterns.

**Fig 1:**
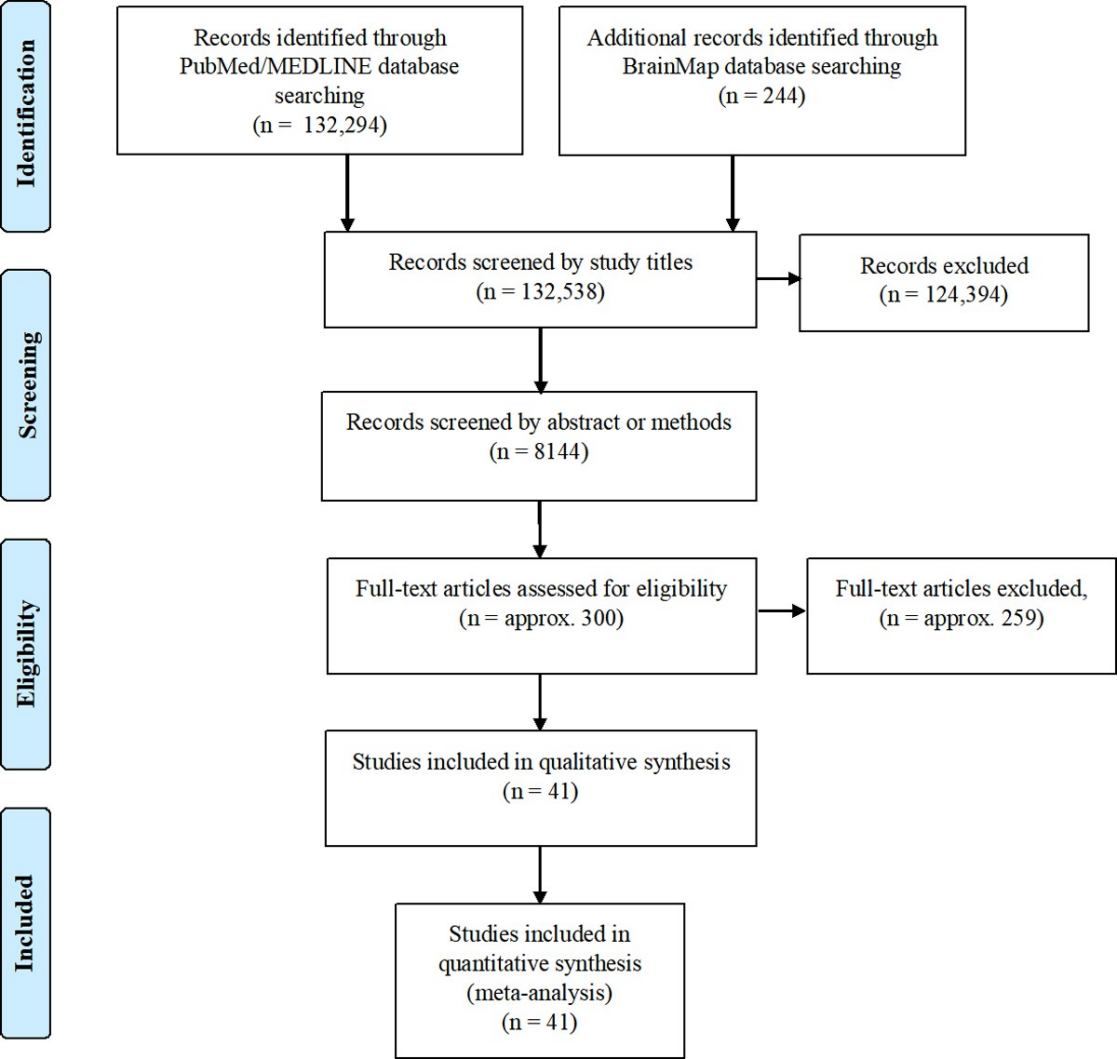
Flow diagram of study review.

To perform the ALE meta-analysis, we began by first locating relevant studies. Relevant studies were those that utilized functional brain imaging of healthy subjects listening tasks. We conducted literature searches in Medline and the BrainMap database [42] using a combination of the following: (1) a functional brain imaging modality, including positron emission tomography (PET) and functional magnetic resonance imaging (fMRI) and (2) relevant adjectives related to auditory stimuli. For example, a single search consisted of “Imaging” AND “passive listening” OR “fMRI or functional magnetic resonance imaging” AND “auditory”. The literature search of Medline was performed February 2016 and returned 132,294 papers. The literature search of BrainMap was performed September 14, 2016 and returned 244 studies. To ensure our ability to investigate the specified research questions a subsequent study selection process was done by applying the following inclusion criteria to the studies: (1) subjects were healthy adult participants; (2) The analyses include contrasts against rest or a suitable low-level control condition; (3) peak coordinates of group-level activations were reported; (4) foci activation were available in the form of standardized stereotaxic coordinates in either Talairach or Montreal Neurological Institute (MNI) space; (5) that results from the entire scanned volume were reported; and (6) data were available as of September 2016. An effort was made to obtain unreported coordinates from selected studies meeting all other criteria, however, this effort did not return any results. The subsequent review process was performed in two phases. First, an automated review of study titles was done using the R environment (R Development Core Team, 2008) to remove studies that were not in healthy human subject populations. The automated review removed 8144 papers from the database. Next, reviewers read the abstract and/or methods sections of remaining studies to assess appropriateness using the above inclusion criteria. Fig 1 illustrates the full review process for the meta-analysis. The process yielded 42 experiments that met the criteria for inclusion. A full list of experiments included can be found in Table 1. Experiments included a total of 386 unique subjects, approximately 195 male and 171 female.

**Table 1:** A list of the studies and experiments that were part of our meta-analysis.

*Experiment*	*Subj*	*Foci*	*Comparison*	*Instructions*	*Music Type*	*Musical Training*	*Age*	*Male*	*Handedness*
Alluri et al., 2012 [56]	11	215	Listen vs. rest	Remain still and to relax while listening to the musical stimulus and to maintain their gaze on the screen	Modern tango (*Adios Nonino by Astor Piazzolla*)	Mean years of music training 16.1 ± 6 SD	Mean age 23.2 ± 3.7 SD	6 (55%)	Unknown
Baumgartner et al., 2006 [57]	9	27	Listen and look at picture vs. fixation baseline	Instructed the subjects to place themselves into the same mood as expressed by the presented emotional stimuli	Emotional classical orchestra music	Unknown	Mean age 24.8; range 21–30	0 (0%)	Right
Blood et al., 1999 [58]	10	4	Listen vs. baseline (acoustically matched noise bursts)	Instructed to listen carefully. After the scan, subjects used a bipolar rating scale to rate emotional valence and intensity of stimuli.	Novel emotional music with varying dissonance	No more than amateur training	Unknown	5 (50%)	Right
Blood et al., 1999 [58]	10	8							
Brown et al., 2004 [60]	10	21	Listen vs. rest	Instructed to listen attentively to the music with their eyes closed without making any movement or response.	Wordless, instrumental rembetika style songs (unfamiliar to participants)	Nonmusicians	Mean age 33.8; range 21–51	5 (50%)	Right
Brown et al., 2007 [59]	11	57	Listen vs. rest; Listen and discrimination task vs. control (button press)	Melody listening: listen with eyes closed. Discrimination task: Listen and button press.	Piano melodies and harmonies, primarily adapted for this work.	University music education majors with a mean of 5.0 years of formal music instruction in voice or instrument. Having had an average of 12.3 years of involvement in musical production.	Mean age 24.6; range 19–46	5 (45%)	Right
Caria et al., 2011 [61]	14	20	Listen vs. silent control	Instructed to passively attend to music.	Instrumental pieces	Nonmusicians	Mean age 24.3 ± 3.02 SD	6 (43%)	Unknown
Chen et al., 2008; Exp 1 [5]	12	18	Listen with anticipation vs. silent baseline	Listened attentively	Rhythmic music	Nonmusicians	Mean age 23.83; range 20–32	6 (50%)	Right
Chen et al., 2008; Exp 2 (A) [5]	12*	17	Listen with anticipation vs. silent baseline	Passively listen	Rhythmic music	Nonmusicians	Mean age 24; range 19–34	6 (50%)	Right
Chen et al., 2008; Exp 2 (B) [5]	12*	9	Passive Listen vs. silent baseline	Passively listen	Rhythmic music	Nonmusicians	Mean age 24; range 19–34	6 (50%)	Right
Demorest et al., 2010 [62]	16	15	Listen vs. rest	Listen, followed by memory test.	Three music examples from the Western classical tradition, three examples from the Turkish classical tradition and three examples from the Chinese classical tradition	<1 year of private music lessons and <3 years of ensemble (e.g., choir and orchestra) participation	Mean age 28.6 years with a range of 20.1–45.1 years	8 (50%)	Right
Dobek et al., 2014 [63]	12	33	Listen vs. baseline	Administered pain (thermal stimulation)	Self-selected by participants	Non-musicians	Range 18–40 years	0 (0%)	Unknown
Flores-Gutierrez et al., 2007 [64]	19	7^**^	Music–noise	Instructed to remain attentively focused on the auditory stimuli as their only task	Complex emotional musical pieces	No formal musical training	Mean age 25 (SD = 3.05)	11 (58%)	Right
Grahn et al., 2007 [6]	27	12	Music—rest	Instructed not to move any part of their body during presentation of the rhythms, followed by response given by button press to rhythm discrimination task	Rhythmic sequences	Fourteen out of 27 had musical training, defined as over 5 years of formal musical training and current regular musical activity and 13 had no musical training (reported no formal musical training or musical activities).	Mean age 24.5; range 19–38	19 (70%)	Right
Habermeyer et al., 2009 (A) [65]	16*	8	Listen vs. silent baseline	Watch silent movie without paying attention to the presented sounds	Deviant melodic patterns	8 trained lifelong musicians; 8 nonmusicians	Mean age 44.5 ± 9.9 years	14 (88%)	
Habermeyer et al., 2009 (B) [65]	16*	3	Listen vs. silent baseline	Watch silent movie without paying attention to the presented sounds	Standard melodic patterns	8 trained lifelong musicians; 8 nonmusicians	Mean age 44.5 ± 9.9 years	14 (88%)	Unknown
Heine et al., 2015 [66]	8	19	Music vs. baseline sounds	Instructed to keep their eyes closed, stay awake, avoid any structured thoughts, and listen attentively to the music	Dynamic musical excerpts chosen by loved ones from a list	Unknown	Mean age 26, SD ± 3	4 (50%)	Unknown
Hugdahl et. al., 1999 [67]	12	5	Musical instruments–simple tones	Button press at target sound	Excerpts from musical instruments	Unknown	Range 20–30	12 (100%)	Right
Langheim et al., 2002 [68]	6	4	Passive listening vs. Rest	Passive listening	Classical music (Vivaldi’s Concerto in G minor, Bach's Suite in C major, part 2, Partita 2 and Partita 3)	At least 15 years of musical experience (two violinists, one pianist and three cellists); mean length of study 19.6 years, range 15–26 years	Mean age 27; range 22–32	2 (33%)	Right
Leaver et al., 2009; (A) [69]	10	9	Familiar and unfamiliar music	Subjects were instructed to attend to the stimulus being presented and to imagine, but not vocalize, the subsequent melody	Short piano melodies constructed for this experiment	At least 2 years musical experience (mean = 6.5, sd = 4.17)	Unknown	Unknown	Unknown
Leaver et al., 2009 (B) [69]	9	3	Familiar and unfamiliar music	Subjects were instructed to attend to the stimulus being presented and to imagine, but not vocalize, the subsequent melody	Short piano melodies constructed for this experiment	Nonmusicians	Unknown	6 (67%)	Unknown
Mirz et al., 1999 [70]	5	7	Music–baseline	Subjects were asked to listen to the presented sounds without performing any semantic, phonological, temporal, intensity, or pitch analysis	Classical music (W.A. Mozart, Piano Concerto No. 21, 65 dB SPL)	Unknown	Mean age 34; range 24–50	2 (40%)	Right
Morrison et al., 2003 (A) [71]	6*	3	Music vs. rest	Following the scan subjects completed a poststudy recognition test	3 Baroque Western examples	Trained professional violinists and violists	mean age 38.3 years	2 (33%)	2 left handed, 4 right handed
Morrison et al., 2003 (B) [71]	6*	3	Music vs. rest	Following the scan subjects completed a poststudy recognition test	3 Chinese examples	Trained professional violinists and violists	mean age 38.3 years	2 (33%)	2 left handed, 4 right handed
Morrison et al., 2003 (C) [71]	6*	2	Music vs. rest	Following the scan subjects completed a poststudy recognition test	3 Baroque Western examples	Non-musicians	mean age 34.2 years	2 (33%)	Right
Morrison et al., 2003 (D) [71]	6*	2	Music vs. rest	Following the scan subjects completed a poststudy recognition test	3 Chinese examples	Non-musicians	mean age 34.2 years	2 (33%)	Right
Ohnishi et. al., 2001 (A) [72]	14	5	Music vs. rest	Instructed to passively listen to music	Italian concert BMV 989 by J.S. Bach	>12 years of 4–8 h of training per day) with AP (n = 10) or relative pitch (n = 4)	Range 20–27	2 (14%)	Right
Ohnishi et. al., 2001 (B) [72]	14	4	Music vs. rest	Instructed to passively listen to music	Italian concert BMV 989 by J.S. Bach	Nonmusicians (no formal education musical and never played an instrument)	Range 21–27	2 (14%)	Right
Rogalsky et. al., 2011 [73]	20	5	Melodies vs. rest	Passive listening	Simple novel piano melodies	Twelve participants had some formal musical training (mean years of training = 3.5, range 0–8)	Mean age 22.6 years; range 18–31	9 (45%)	Right
Satoh et al., 2001 [75]	9*	8	Music (alto) vs. baseline	Subjects were asked to listening to and concentrate on the tone of the alto part of the harmony, and make a sign when they heard the tonic tone	3 fairly unknown motets; musical pieces of harmonious style with four vocal parts, composed by Anton Bruckner.	Musicians (music students)	Mean age 21.8 years; range 21–28	9 (100%)	Right
Satoh et al., 2001 [75]	9*	10	Music (harmony) vs. baseline	Subjects were asked to listen to the melody as a whole, and make a sign upon hearing the minor chord	3 fairly unknown motets; musical pieces of harmonious style with four vocal parts, composed by Anton Bruckner.	Musicians (music students)	Mean age 21.8 years; range 21–28	9 (100%)	Right
Satoh et. al., 2003 [76]	11*	7	Music (soprano) vs. baseline	Subjects were asked to listen to the soprano part of the harmony, and make a sign when they regarded a tonal sequence as one phrase	Three new musical pieces of harmonious style with three vocal parts	Nonmusicians (no formal musical education or training)	Mean age 21.2 years; range 20–30	11 (100%)	Right
Satoh et. al., 2003 [76]	11*	10	Music (harmony) vs. baseline	Subjects were asked to listen to the melody as a whole, and make a sign upon hearing a dissonant chord	Three new musical pieces of harmonious style with three vocal parts	Nonmusicians (no formal musical education or training)	Mean age 21.2 years; range 20–30	11 (100%)	Right
Satoh et. al., 2006 [74]	10*	16	Music (familiarity) vs. baseline	Subjects were asked to listen to the melodies and then judge whether the melody was familiar	33 melodies (27 melodies were well-known old Japanese nursery songs)	Nonmusicians (no formal musical education or training)	Mean age 21.6; range 20–28	10 (100%)	Right
Satoh et. al., 2006 [74]	10*	13	Music (alteration-detecting task) vs. baseline	Subjects were asked to listen to the same melodies and detect the altered notes by making a sign	33 melodies (27 melodies were well-known old Japanese nursery songs)	Nonmusicians (no formal musical education or training)	Mean age 21.6; range 20–28	10 (100%)	Right
Schmithorst, 2005 [77]	15	30	Melodies—random tones	Passive listening	30 s of an unharmonized popular melody, followed by 30 s of tones of random frequency and duration, followed by 30 s of the previous melody, harmonized using triads an octave below	7 out of 15 received prior formal musical training, receiving formal instruction, continuously from early childhood (8 years old) throughout adolescence	Mean age 37.8 ± 15.2 SD	11 (73%)	Unknown
Toiviainen et al., 2014 [78]	15	38			Comprised the B-side of the album Abbey Road by The Beatles (1969).	Unknown	Mean age 25.7 ± 5.2 SD	10 (67%)	Right
Trost et al., 2011 [79]	15	20	Music vs. random tones	Subjects were asked to listen closely and provided a rating of emotional feeling following the music piece	Emotional classical music	No professional music experience	Mean age 28.8 +- 9.9	8 (53%)	Right
Tsai et al., 2010 [49]	12*	7	Music—baseline	Subjects were asked to passively listen to unlearned percussion music	Sichuan opera percussion music, Beijing opera percussion music, syllable representation of Beijing opera percussion music, and Taiwanese opera tunes played by the erhu	Music training for more than 4 years	Range: 20–26	2 (17%)	Right
Tsai et al., 2010 [49]	12*	7	Music—baseline noise	Subjects were asked to listen and hum covertly along to learned percussion music	Sichuan opera percussion music, Beijing opera percussion music, syllable representation of Beijing opera percussion music, and Taiwanese opera tunes played by the erhu	Music training for more than 4 years	Range: 20–26	2 (17%)	Right
Tsai et al., 2010 [49]	12*	7	Music—baseline noise	Subjects were asked to listen and hum covertly along to the verbalized syllable representation of learned percussion music	Sichuan opera percussion music, Beijing opera percussion music, syllable representation of Beijing opera percussion music, and Taiwanese opera tunes played by the erhu	Music training for more than 4 years	Range: 20–26	2 (17%)	Right
Tsai et al., 2010 [49]	12*	7	Music—baseline noise	Subjects were asked to listen and hum covertly along to the verbalized syllable representation of learned melodic music	Sichuan opera percussion music, Beijing opera percussion music, syllable representation of Beijing opera percussion music, and Taiwanese opera tunes played by the erhu	Music training for more than 4 years	Range: 20–26	2 (17%)	Right

* The same pool of participants was used for separate analysis/study protocols. These were considered separate experiments for the purposes of this meta-analysis because analyses were performed separately and/or the dependent variable was altered between conditions.

** The published data was missing one z coordinate. An attempt was made to contact the authors, however, we were unable to obtain the missing information.

Coordinates (X, Y, Z) for selected studies were recorded and, where necessary, transformed to Talairach space. Coordinates from individual studies were transferred to a text file formatted for analysis in GingerALE 2.3.6 (http://www.brainmap.org/ale/; Research Imaging Center, University of Texas, San Antonio, TX). These were transferred either using brainmap’s Sleuth software (if the studies were located in the brainmap database), which outputs coordinates in the correct format for GingerALE, or were transferred individually by hand. The ALE meta-analysis was carried out in GingerALE. The ALE procedure was as follows: (1) model of single-study activation foci as peaks of three-dimensional Gaussian probability densities with subject-based full-width at half-maximum values [43]; (2) summation of probability densities to produce a statistical map estimating the likelihood of activation at each voxel; (3) thresholding of this ALE map based on the null hypothesis of a uniform distribution of foci; (4) correcting for multiple comparisons by family-wise error thresholding. Resulting statistical maps show clusters where convergence between foci is greater than would be expected by chance. Statistical maps were thresholded using cluster-level family-wise error correction P<0.05 (cluster-forming threshold voxel-level P<0.001).

We split the data into separate studies that used either musicians only or nonmusicians only, with the intention of performing a contrast analysis between the two groups. Unfortunately, there were too few studies in these groups individually (14 experiments in each group), so we were unable to complete this contrast.

## Results

**Fig 2:**
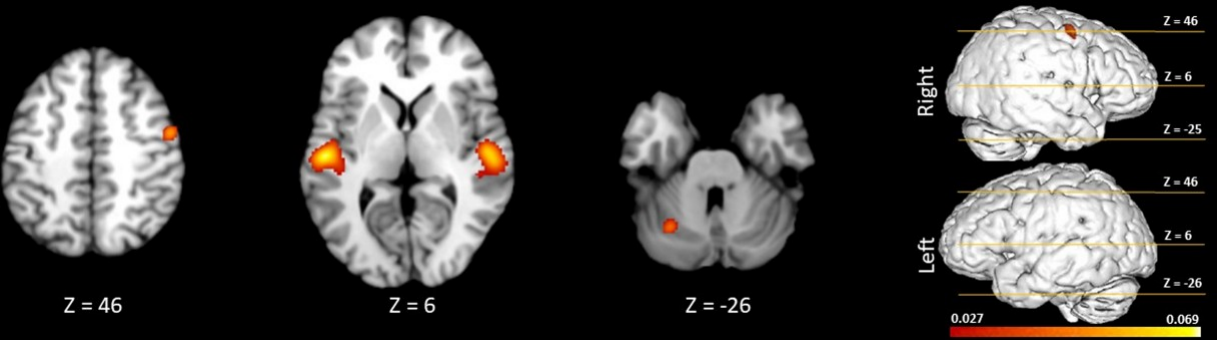
Significant clusters from meta-analysis of passive listening tasks in healthy volunteers (family-wise error correction (P<0.05)). The 3D brain is shown to indicate slice levels.

Fig 2 shows the activations during passive listening, demonstrating the common brain network underlying music perception. Talairach coordinates for these ALE foci are presented in Table 2. Activations were seen in the bilateral superior temporal gyrus, transverse temporal gyrus, insula, pyramis, bilateral precentral gyrus, and bilateral medial frontal gyrus. As shown in Fig 2, there was activation in the left and right premotor cortex (BA 6), right primary motor cortex (BA 4), and the left cerebellum.

**Table 2:** Talairach coordinates for voxel clusters.

Area		BA	Conjunction			
			x	y	z	ALE
**Temporal Lobe**						
**Superior Temporal Gyrus**	R	22	52	-16	6	0.061329
	R	22	52	-6	-4	0.054407
	L	41	-52	-18	6	0.069281
	L	41	-42	-34	12	0.033931
**Frontal Lobe**						
**Precentral Gyrus**	R	4	50	-4	46	0.052819
**Anterior Lobe**						
**Cerebellum (Culmen)**	L		-28	-60	-26	0.045433

The Talairach coordinates of the significant ALE clusters are presented for the conjunction of passive listening (p < 0.05, FWE). The ALE values for the conjunction represent the minimum ALE value from the passive listening ALE maps. The ALE values shown are the true values times 10^^-3^. BA, Brodmann area.

An inspection of Table 3 reveals that Cluster 1 is centered over the right primary auditory cortex, and spans from BA 22 and BA 41/42 (primary and secondary auditory cortices) in the right hemisphere to BA 6 (right premotor cortex). Likewise, in the left hemisphere, cluster 2 is centered over the left primary auditory cortex, and spans from BA 22 and BA 41/42 (primary and secondary auditory cortices) in the left hemisphere to BA 6 (left premotor cortex). Cluster 3 reveals motor system activation in the right hemisphere, centered in right premotor cortex and spanning from premotor to primary motor cortex. Finally, cluster 4 is located in the left cerebellum. Fig 2 depicts the activation patterns seen bilaterally for a range of *z* values.

**Table 3:** Contributing foci and study making up each voxel cluster.

Cluster	Label	Total # of Foci	Cluster Size	BA	Studies Contributing to Cluster
**1**	Right Superior Temporal Gyrus	75	6336 mm^3^	22	19 foci from Alluri, 2012 3 foci from Baumgartner, 2006 5 foci from Brown, 2004 5 foci from Brown, 2007 2 foci from Chen, 2008: Experiment 1 2 foci from Chen, 2008: Experiment 2 A 1 foci from Chen, 2008: Experiment 2 B 1 foci from Dobek, 2014 1 foci from Flores-Gutierrez, 2007 1 foci from Habermeyer, 2009 1 foci from Habermeyer, 2009 1 foci from Heine, 2015 1 foci from Hugdahl, 1999 1 foci from Langheim, 2002 1 foci from Leaver, 2009 1 foci from Leaver, 2009 1 foci from Mirz, 1999 1 foci from Morrison, 2003 A 1 foci from Morrison, 2003 B 1 foci from Morrison, 2003 C 1 foci from Morrison, 2003 D 2 foci from Ohnishi, 2001 A 2 foci from Ohnishi, 2001 B 2 foci from Rogalsky, 2011 B 1 foci from Satoh, 2006 1 foci from Schmithorst, 2005 4 foci from Toiviainen, 2014 2 foci from Trost, 2011 3 foci from Tsai, 2010 A 2 foci from Tsai, 2010 B 2 foci from Tsai, 2010 C 3 foci from Tsai, 2010 D
**2**	Left Superior Temporal Gyrus	62	5248 mm^3^	41	15 foci from Alluri, 2012 1 foci from Baumgartner, 2006 1 foci from Blood, 1999 2 foci from Blood, 1999 1 foci from Brown, 2004 7 foci from Brown, 2007 1 foci from Chen, 2008: Experiment 1 1 foci from Chen, 2008: Experiment 2 A 3 foci from Chen, 2008: Experiment 2 B 2 foci from Demorest, 2010 1 foci from Flores-Gutierrez, 2007 1 foci from Grahn, 2007 1 foci from Habermeyer, 2009 1 foci from Habermeyer, 2009 1 foci from Heine, 2015 1 foci from Hugdahl, 1999 1 foci from Langheim, 2002 1 foci from Leaver, 2009 1 foci from Leaver, 2009 1 foci from Mirz, 1999 1 foci from Morrison, 2003 A 1 foci from Morrison, 2003 B 1 foci from Morrison, 2003 C 1 foci from Morrison, 2003 D 1 foci from Ohnishi, 2001 A 1 foci from Ohnishi, 2001 B 2 foci from Rogalsky, 2011 B 3 foci from Toiviainen, 2014 2 foci from Tsai, 2010 A 1 foci from Tsai, 2010 B 2 foci from Tsai, 2010 C 2 foci from Tsai, 2010 D
**3**	Right Precentral Gyrus	12	824 mm^3^	4	1 foci from Alluri, 2012 1 foci from Baumgartner, 2006 1 foci from Brown, 2007 2 foci from Caria, 2011 1 foci from Chen, 2008: Experiment 1 1 foci from Chen, 2008: Experiment 2 A 1 foci from Chen, 2008: Experiment 2 B 1 foci from Grahn, 2007 1 foci from Tsai, 2010 B 1 foci from Tsai, 2010 C 1 foci from Tsai, 2010 D
**4**	Left Anterior Lobe	15	760 mm^3^		8 foci from Alluri, 2012 1 foci from Brown S, 2007 1 foci from Caria, 2011 1 foci from Chen J L, 2008: Experiment 1 1 foci from Chen J L, 2008: Experiment 2 B 1 foci from Grahn J A, 2007 1 foci from Tsai, 2010 C 1 foci from Tsai, 2010 D

## Discussion

We found evidence for consistent activation of various regions of the brain during passive music listening. As expected, our results showed activation in the primary and secondary auditory areas bilaterally. This is consistent with the existing literature showing that these areas are the critical regions of cortex for processing incoming auditory information [44]. Other activated areas included both right primary motor cortex, right and left lateral premotor cortex, and left cerebellum. We discuss in turn the implications for each of these findings below.

### Activation of premotor cortex

We were unable to pinpoint activation to any further subregions of lateral PMC (i.e., dorsal or ventral), as the activation pattern could be consistent with either dorsal or ventral PMC. The average coordinates for these regions overlap in such a way that neither can be ruled out. This means that premotor involvement could be via dorsal, ventral, or both. The activation of PMC in the present analysis is consistent with both ASAP and the HAPEM framework. However, because we do not know whether this activation is localized to ventral or dorsal PMC, it is unclear if this activity reflects involvement of the dorsal stream, or potentially the action observation network that recruits vPMC for action simulation. Also, given that these clusters only represent aggregate BOLD activation, we do not have insight into the temporal dynamics of this activity, which will be crucial for inference about its origin.

### Activation of primary motor cortex

M1 activity could reflect either an excitatory or inhibitory contribution, as the BOLD signal does not differentiate the two. Vigneswaran et al. [45] report that while many M1 neurons are active during action observation and thus classified as mirror neurons [46], M1 neurons directly connecting to spinal circuitry and thus contributing to observed action are suppressed during the observation of action, in order to prevent explicit action. Simulative properties of mirror neurons have also been confirmed in response to auditory sounds [47]. Thus, either excitatory, inhibitory, or both could give rise to activation of M1 during passive music listening. Some theories of mirror neuron activity [48] additionally claim that the mirror neuron network uses active inference during the perception of observed actions using predictive mechanisms.

Further examination of the studies that contributed to the right premotor/primary motor activation cluster reveals that a number of them used tasks that were not passive listening in the same way as passive background listening. For example, Chen et al. [5] required in some experimental conditions for participants to anticipate later tapping to the beat in subsequent trials, which may recruit motor planning regions during the listening task. Grahn and Brett [6] asked their participants to determine whether the rhythms of two stimuli were the same or different, which may recruit motor areas to assist with the detection task. Finally, Tsai et al. [49] asked participants in some of their tasks to covertly hum along with the music that they were hearing, which may recruit motor areas for subvocalization. Therefore, more work should be done to decisively conclude whether motor areas are recruiting during background listening, in addition to passively listening for properties of the music while remaining still.

### Activation of cerebellum

Many studies contributed to the cluster indicating left cerebellum. The activation of PMC and cerebellum during music listening supports predictive theories of the motor system, such that the cerebellum might provide the predictive component in a forward model of the upcoming sensory consequences. The cerebellum may be providing an inverse model for mapping sensory input to the simulated movement that would give rise to that sensation. An investigation of the temporal dynamics of communication among these regions can again provide further insight into the mechanism.

### Lack of activation in SMA/pre-SMA and basal ganglia structures

While quite a few studies did report activation of SMA and pre-SMA, we did not find corresponding activation in our meta-analysis. We also failed to find evidence of basal ganglia activation. One potential reason for this discrepancy may be that another process on top of passive listening is needed to engage these regions. The generally agreed upon roles of both SMA and the basal ganglia are of sequential learning and timing [31, 50, 51]. Because these are properties of the majority of music, this region is likely to be recruited in many musical contexts. However, without directly listening for these properties of the music, it appears that automatic SMA and basal ganglia activation is not prevalent. Looking more closely at those experiments that explicitly report SMA/pre-SMA activation, they do appear to have a musical beat component to them, which relies on the underlying sequential and timing properties of the music. Bengtsson et al [4] encouraged participants to focus on rhythmic properties of the music, as did Chen, Penhume and Zatorre [5] and Grahn and Brett [6]. Baumann et al. [1] required subjects to do a counting task during passive listening as a distractor, which may have resulted in SMA activation. Experiments showing basal ganglia activation also appear to involve a beat detection task [29].

It also may be the case that activation of SMA/pre-SMA is only prevalent in musically-trained individuals, who are more likely to attend to and perceive the structural aspects of the music due to their background training. Baumann et al. [1] report increased activation of both pre-SMA and SMA in musicians compared to nonmusicians, as did Bangert [3] for SMA activation. Participants in Meister et at [2] showing SMA activation were all musicians. Thus, it appears SMA activation is likely due to either a trained musical background and/or a focus on the rhythmic properties of music.

These results show that the passive perception of music engages a large and complex network of brain regions. This includes activation of areas in the motor system proper. Activation of the cerebellum and primary and premotor cortices suggests that perceived music is partly processed in areas typically considered as important for action-relevant information only. Recruitment of premotor areas during music listening supports many theories of motor involvement during perceptual tasks [11, 17, 19]. The idea of shared neural resources for tasks with underlying computational similarities has gained recent theoretical and grounded neurobiological support [16]. Most current theories suggest that perceptual processing involves the same kinds of temporal prediction involved in action, making a shared circuit useful for action-based and perception-based processing. An alternative (or potentially compatible) hypothesis is that involvement of PMC reflects the process of simulation [35], where the motor system underlies simulation of the actions required to create the observed sensory information. Our findings are consistent with both of these theoretical frameworks, though it does not provide any insight for distinguishing which theory best fits the data, as this meta-analysis only tells us which areas are active at some point in the process. This work supports the currently merging conceptualizations of action and perception [14, 48].

One limitation of the present meta-analysis was that we were unable to obtain data from contacted authors for studies that did not report all of the observed brain activations. It is possible that unpublished or unreported activations may have biased our results toward reporting motor and auditory areas, as studies that do not find activation in these areas of interest are less likely to be reported. This inability to obtain unavailable data also likely contributed to our inability to obtain enough studies for the musician/nonmusician contrast. Further exploration of differential activation in musicians relative to nonmusicians is important for advancing this work. Musicians exhibit plasticity-induced changes perceptual and motor abilities, as well as changes in structural and functional neuronal connectivity [52–55]. In particular, we believe that musicians passively listening to music should also recruit supplementary motor cortex, and might show greater activation of the cerebellum, which has a larger volume in musicians [54]. Another interesting avenue to pursue is to run more studies that directly compare different types of music listening tasks. For instance, we might compare background listening to listening in anticipation of some movement to listening for particular musical features, such as rhythm or grooviness. This will incorporate context-dependent music listening, which may reveal that different (but likely highly overlapping) networks are recruited in separate contexts. An additional limitation of this approach is that while we can identify which brain areas are active at some point during the music listening task, the BOLD signal cannot tell us anything about the temporal dynamics of the process. Complementary methods, such as EEG, should be used along with imaging data to investigate the functional connectivity among these music listening networks. This will also allow us to determine which of the existing theories fit best with the data.

In summary, this study adds support to the idea that motor planning activity serves not only to help us move but is recruited for music perception even in the absence of movement. Further exploration will elucidate the functional purpose of this recruitment, as well as why and how different music listening contexts seem to engage slightly different networks. An understanding of the auditory-motor interactions underlying music perception could explain a growing number of findings suggesting an important link between music perception and the action systems of the brain.

## Supporting information

S1 File Final dataset as a NIFTI file. (NII)

S2 File Completed PRISMA checklist for meta-analysis. (DOC)
